# Delayed Destructive Osteoarticular Infection Caused by Pasteurella multocida Following Misleading Initial Clinical Improvement: A Case Report

**DOI:** 10.7759/cureus.113134

**Published:** 2026-07-21

**Authors:** Taieb Iheb, Khairi Saibi, Doha Laamarti, Lilya Sellami, Mouna Ounaies

**Affiliations:** 1 Hand Surgery, Mohamed Kassab Institute of Orthopaedics, Tunis, TUN; 2 Plastic and Hand Surgery, University of Tunis, Tunis, TUN; 3 Hand Surgery, Mohamed Kassab Institute of Orthopedics, Tunis, TUN

**Keywords:** arthrectomy, feline bites, hand infection, osteoarthritis, pasteurella multocida

## Abstract

A 35-year-old woman presented with a digital infection after exposure to cat saliva without a clearly identified bite wound. She underwent surgical debridement and received antibiotic therapy, with apparent initial clinical improvement. Despite favorable early evolution, she returned four weeks later with pain, swelling, and stiffness of the affected finger. Further evaluation revealed a destructive infection involving the proximal interphalangeal joint, requiring additional surgical management.

This case highlights the potential for delayed deep-seated infection following animal-related hand injuries, even in the absence of an obvious bite and despite initial clinical improvement. Close follow-up and early consideration of osteoarticular involvement are essential to prevent functional impairment.

## Introduction

*Pasteurella multocida* is a Gram-negative coccobacillus that forms part of the oral and respiratory flora of cats and dogs [1]. It is frequently responsible for soft tissue infections of the hand following animal bites or scratches, with cat bites being the most common mode of transmission [2]. These infections generally respond favorably to appropriate antibiotic therapy and surgical debridement when required. However, deeper infections involving osteomyelitis and septic arthritis may occur, particularly when diagnosis or adequate treatment is delayed.

Although most reported osteoarticular infections are associated with direct inoculation following animal bites or puncture wounds [3], delayed destructive osteoarticular complications after non-bite exposure to cat saliva remain uncommon and poorly described.

We report the case of a digital *Pasteurella multocida* infection following cat saliva exposure, with delayed osteomyelitis and septic arthritis despite initial clinical improvement. This case highlights the importance of close follow-up and clinical vigilance in animal-related hand infections, even when the early clinical course appears favorable.

## Case presentation

A 35-year-old right-handed woman with no significant past medical history presented to the emergency department with pain in the right middle finger 10 days after an untreated wound. She had self-administered antibiotics prior to admission. The patient reported that the wound had been licked by her domestic cat and denied any cat bite or deep puncture injury.

Initial clinical examination showed inflammatory signs, including redness and tenderness over the proximal interphalangeal (PIP) joint, associated with a subcentimetric wound opposite the PIP joint of the middle finger and purulent discharge, without fever or general deterioration (Figure 1). 

**Figure 1:**
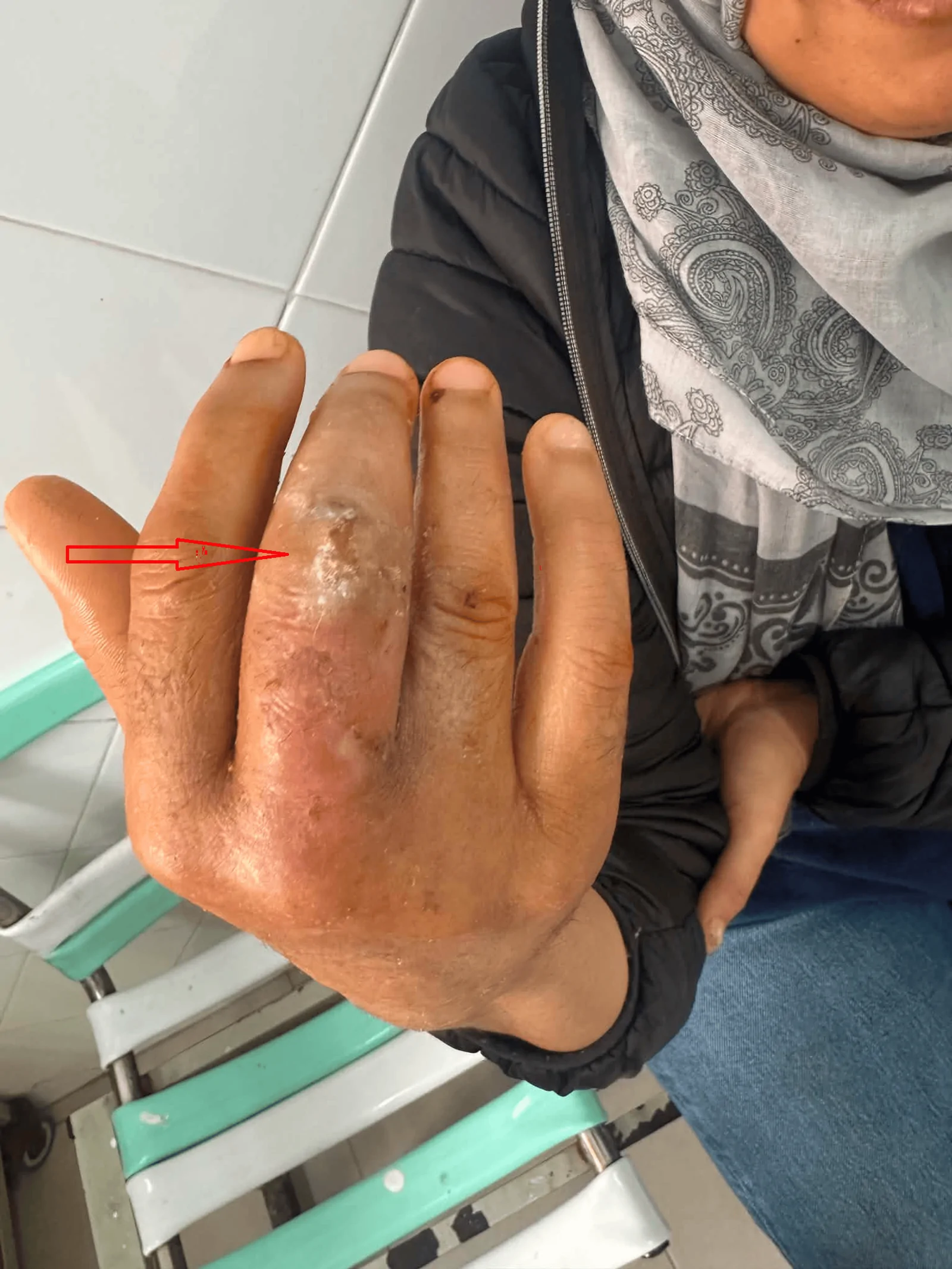
Local examination 10 days after the initial wound. The figure shows erythema and tenderness over the proximal interphalangeal joint, associated with a subcentimetric wound opposite the PIP joint of the middle finger and purulent discharge (red arrow).

Laboratory investigations demonstrated a moderate inflammatory syndrome (Table 1).

**Table 1:** Laboratory findings at initial presentation.

Parameter	Result	Unit	Reference Range
White blood cell count	9.27	10⁹/L	4-10
Neutrophils	9000	cells/mm³	1700-7000
C-reactive protein (CRP)	22	mg/L	<20

Initial X-rays showed no obvious bone abnormalities (Figure 2).

**Figure 2:**
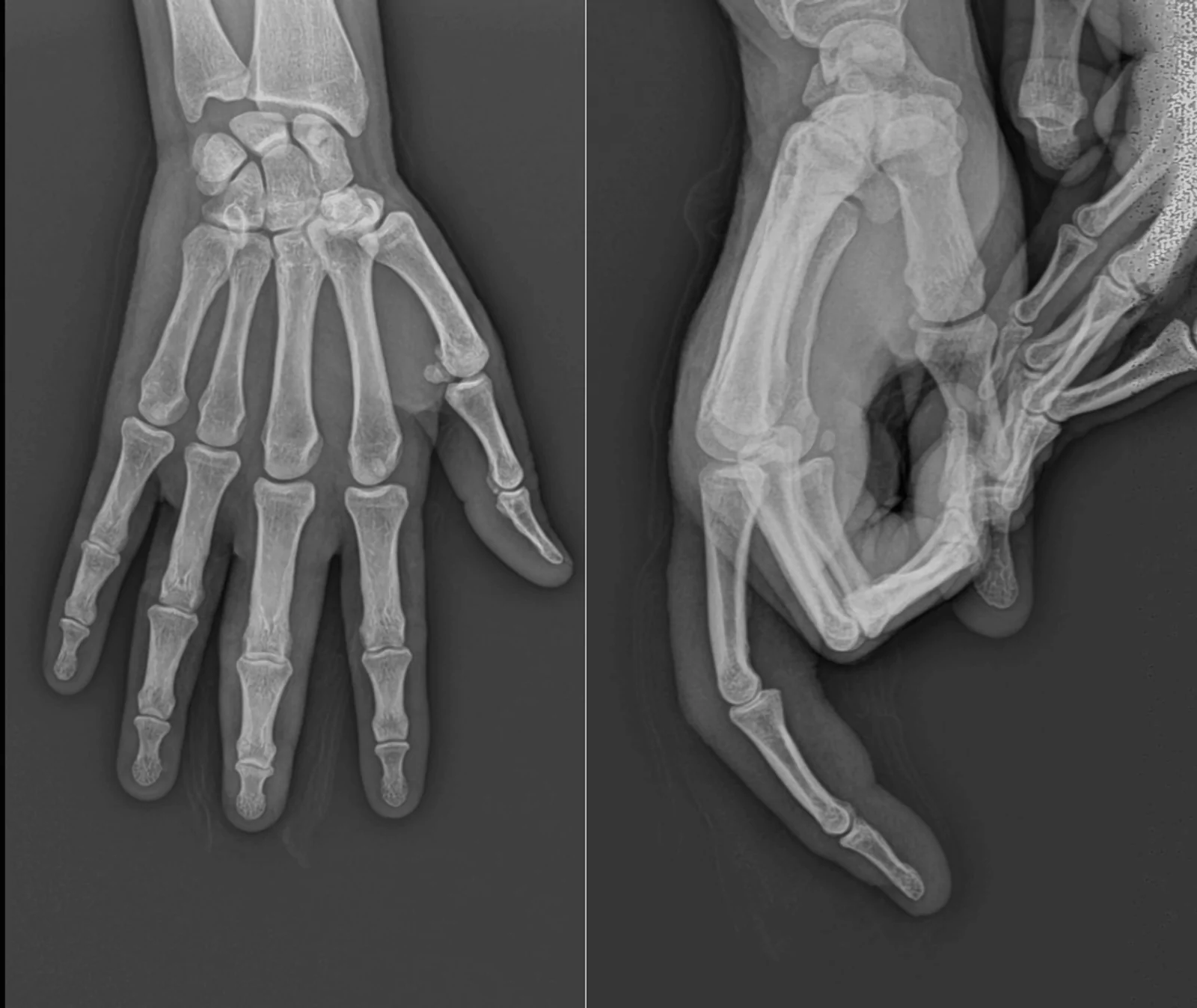
Initial X-rays of the hand showing no abnormalities.

The patient was admitted to the plastic surgery department, where urgent surgical debridement was performed because of the articular location of the lesion. Intraoperative findings were consistent with stage II septic arthritis of the proximal interphalangeal joint, justifying placement of a Beaubourg-type external fixator, followed by empirical intravenous antibiotic therapy with amoxicillin-clavulanic acid for 10 days [4] pending bacteriological results, which revealed multisensitive Pasteurella multocida infection (Table 2). 

**Table 2:** Bacteriological culture results and antimicrobial susceptibility profile.

Specimen Collected	Culture Result	Isolated Pathogen	Susceptible Antibiotics
Deep pus specimen obtained during surgical debridement	Positive bacterial growth	Pasteurella multocida	Amoxicillin–clavulanic acid, amoxicillin, cefotaxime, levofloxacin, nalidixic acid, penicillin G, trimethoprim–sulfamethoxazole, tetracycline

The initial course was favorable, with marked reduction in pain and edema over 10 days, and the patient was discharged on oral amoxicillin-clavulanic acid (1 g three times daily) for an additional four weeks. The total duration of antibiotic therapy was therefore six weeks.

However, four weeks later, the patient developed recurrence of pain, stiffness, and swelling of the proximal interphalangeal joint, associated with cutaneous fistulization and purulent discharge (Figure 3). 

**Figure 3:**
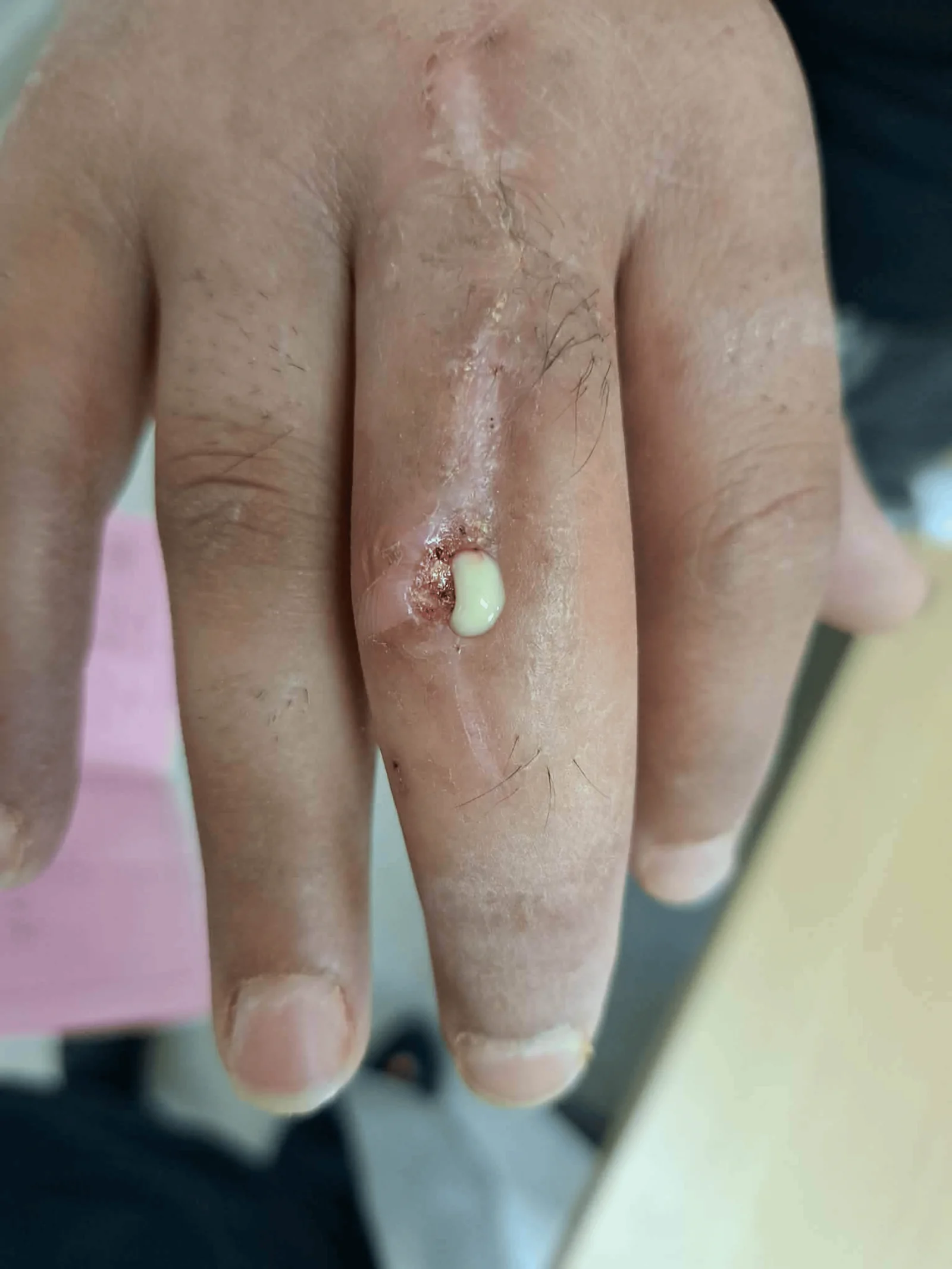
Clinical presentation at recurrence. Local examination prior to revision surgery showing cutaneous fistulization with purulent discharge.

Follow-up X-rays demonstrated joint space narrowing associated with geodes suggestive of PIP osteoarticular infection (Figure 4).

**Figure 4:**
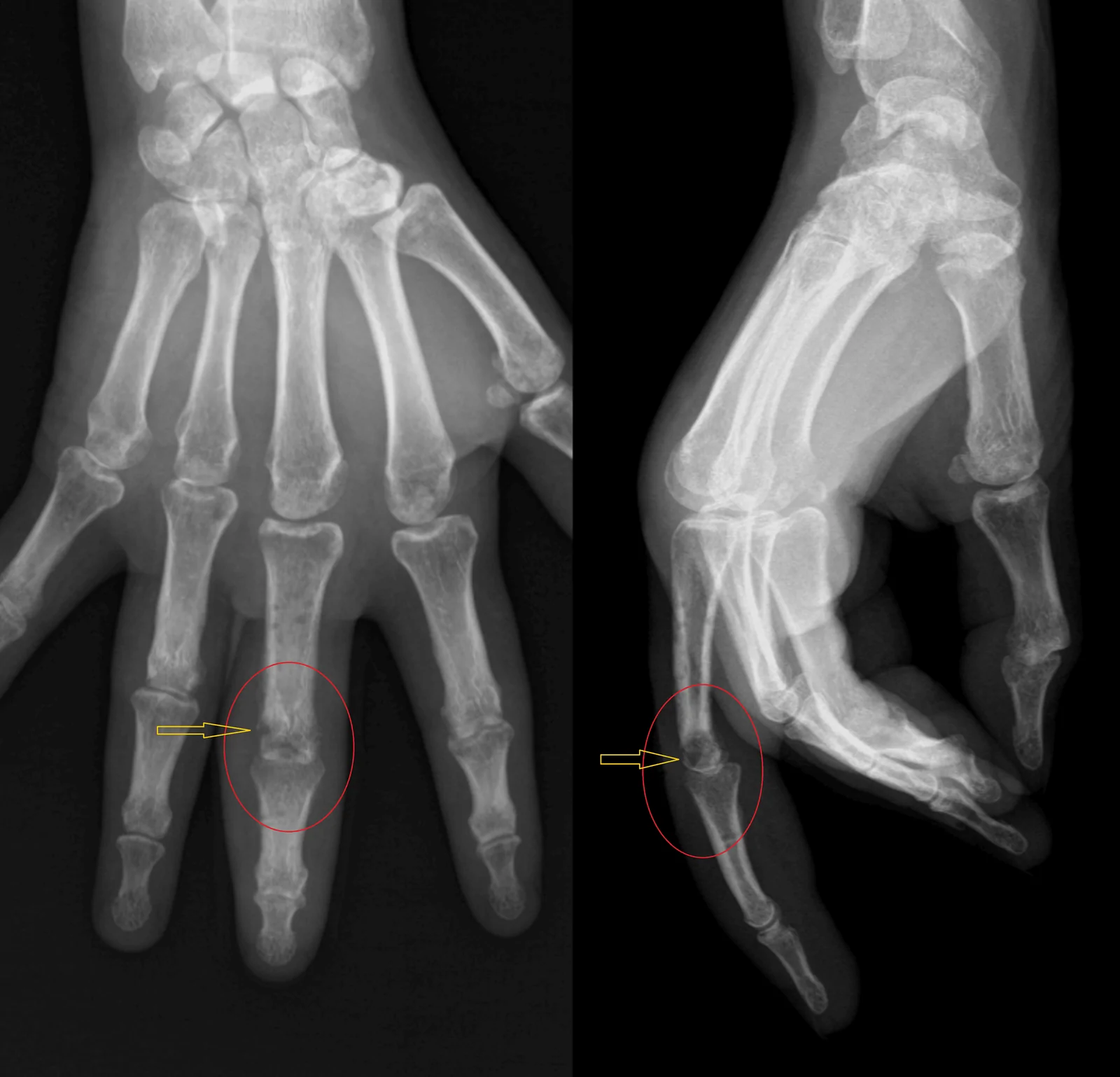
Radiographic findings at recurrence. X-rays of the hand showing narrowing of the proximal interphalangeal (PIP) joint space, with a geode-like lesion (yellow arrow) of the head of the proximal phalanx (P1), consistent with septic arthritis of the PIP joint (red circle).

No MRI or CT imaging was performed during recurrence, as the diagnosis was primarily clinical, supported by the presence of recurrent inflammatory signs, fistulization, purulent discharge, and radiographic evidence of septic joint destruction.

Revision surgery was indicated. Exploration revealed osteochondral destruction of the proximal interphalangeal joint associated with significant chronic inflammatory synovitis, infected granulation tissue, and erosive lesions of the bony articular surfaces consistent with advanced osteomyelitis and septic arthritis. Arthrectomy with distraction using an external fixator was then performed. Intraoperative samples again isolated *Pasteurella multocida*.

At three months' follow-up, the patient achieved complete healing without any clinical or biological evidence of infectious recurrence. The surgical site was fully healed, and the proximal interphalangeal joint was painless. Residual stiffness was observed, with an active range of motion from 20° of extension deficit to 60° of flexion.

Despite this limitation, the patient maintained functional use of the affected finger, with preserved grip and pinch function and no restriction in activities of daily living.

## Discussion

Hand infections caused by *Pasteurella multocida* may initially present with a reassuring clinical course while masking underlying deep tissue involvement. The characteristics of the organism may partly explain this progression, notably the presence of a polysaccharide capsule, an essential virulence factor that enables bacterial survival by evading immune cell activity [5]. Furthermore, digital anatomy, characterized by thin soft tissues and close proximity of tendinous, articular, and osseous structures, particularly on the dorsal aspect of the hand, facilitates rapid spread of infection. Feline bites are punctiform, favoring deep bacterial inoculation that is often clinically underestimated, unlike canine bites, which usually produce extensive tissue lacerations that facilitate drainage [6].

In this clinical case, contamination occurred through salivary contact without an actual bite, a mechanism often underestimated by both patients and practitioners. The initial clinical improvement was probably related to prior antibiotic intake, which masked the initial inflammatory signs and explains the prolonged delay between injury and consultation [3]. Initial imaging was poorly contributive, with delayed appearance of radiological bone lesions.

Resolution of superficial inflammation without eradication of the deep infectious focus, together with bacterial persistence within poorly vascularized structures such as cartilage or subchondral bone, may explain the secondary progression toward osteoarticular involvement.

Although uncommon, osteoarthritic complications caused by *Pasteurella multocida* [7] are well documented and require prompt surgical management [8]. Diagnosis may be delayed because of initially normal imaging findings and transient clinical improvement [9].

To our knowledge, few cases have been reported in the literature describing progression toward delayed destructive osteoarthritis caused by *Pasteurella multocida* infection after transient initial clinical improvement [10,11].

This case highlights the importance of close clinical monitoring of wounds contaminated by cat saliva or bites. Recurrence of pain, swelling, or stiffness should raise suspicion of deep involvement and justify early complementary investigations as well as surgical management when necessary.

## Conclusions

*Pasteurella multocida* infection of the hand may occasionally be complicated by delayed osteoarticular involvement despite an initially favorable clinical course. This case highlights that early clinical improvement does not necessarily reflect complete resolution of a deep-seated infection, particularly following cat-related injuries. Clinicians should remain vigilant and ensure appropriate follow-up, with prompt reassessment of recurrent pain, swelling, or stiffness to identify potential complications and limit functional impairment.
